# Improving the physician-patient cardiovascular risk dialogue to improve statin adherence

**DOI:** 10.1186/1471-2296-10-48

**Published:** 2009-06-30

**Authors:** Linda Casebeer, Craig Huber, Nancy Bennett, Rachael Shillman, Maziar Abdolrasulnia, Gregory D Salinas, Sijian Zhang

**Affiliations:** 1 Outcomes, Inc., 107 Frankfurt Circle, Birmingham, AL, USA; 2 AstraZeneca, 1800 Concord Pike, Wilmington, DE, USA; 3 Consultant, Outcomes, Inc., 107 Frankfurt Circle, Birmingham, AL, USA; 4 University of Alabama, Birmingham, 514 E Ryals Public Health Bldg, Birmingham, AL, USA

## Abstract

**Background:**

The purpose of this study was to evaluate the effectiveness of a patient education program developed to facilitate statin adherence.

**Methods:**

A controlled trial was designed to test the effectiveness of a multifaceted patient education program to facilitate statin adherence. The program included a brief, in-office physician counseling kit followed by patient mailings. The primary end point was adherence to filling statin prescriptions during a 120-day period. Patients new to statins enrolled and completed a survey. Data from a national pharmacy claims database were used to track adherence.

**Results:**

Patients new to statin therapy exposed to a patient counseling and education program achieved a 12.4 higher average number of statin prescription fill days and were 10% more likely to fill prescriptions for at least 120 days (*p *= .01).

**Conclusion:**

Brief in-office counseling on cardiovascular risk followed by patient education mailings can be effective in increasing adherence. Physicians found a one-minute counseling tool and pocket guidelines useful in counseling patients.

## Background

Effective management of lipid levels as risk factors for cardiovascular disease requires long-term adherence to a lipid lowering treatment regimen. However, studies continue to demonstrate lack of persistence in adhering to cholesterol-lowering medicine therapy [1-6]. When individuals at risk for cardiovascular disease do not adhere to prescribed medical regimens, medication benefits are not fully achieved. Only half of individuals continue to take prescribed statins at six months, with further declines in adherence at one year [1]. This finding is consistent in individuals who would benefit from primary prevention with statins, as well as in those who have experienced a cardiovascular event; it is especially prominent in asymptomatic individuals, where such treatment is preventive [6-8].

Understanding and improving the quality of medication management through the patient dialogue is a key issue in adherence by patients treated for chronic diseases [1,9]. The communication of cardiovascular risk has been linked to patients intensifying risk-reducing behaviors and adhering to prescribed therapies [10]. Medication side effects and cost issues that may lead to non-adherence are frequently not discussed with physicians, a communication gap with important quality and safety implications [11,12]. Providers may also not be using the most effective communication strategies when they do ask about medication-taking behavior [9].

A review of randomized controlled trials evaluating the effectiveness of interventions designed to facilitate patient adherence to prescribed medications for chronic health problems found that effective interventions were complex, labor-intensive, and not predictably effective [5]. A recent analysis addressing how adherence to statins could be improved found that simplification of drug regimens, patient information and education and intensified patient care by healthcare professionals showed the most promise [2]. At the present time, no fully effective strategies for adherence are in widespread clinical use; innovative approaches to assist patients in following prescriptions have the potential to enhance the health of the public and reduce healthcare costs [13]. Risk-communication interventions, however, have been associated with improved patient knowledge, perception of risk, and adherence. [14,15] The extra consultation time required for risk counseling and shared decision-making has been associated with benefits to the patient. [15]

When patients begin a new medical regimen, the regimen requires behavioral changes both in implementation and maintenance. The transtheoretical model has been used to describe patients' willingness to make behavioral changes that benefit their health. The model postulates that patients, as they make changes such as adhering to a newly prescribed medical regimen, evolve throughout six stages: precontemplation, contemplation, preparation, action, maintenance, and termination. [16] A review of the use of the transtheoretical model in medication adherence revealed that the model has not been used extensively to investigate medication use, although the model has shown promise as a context for developing interventions to improve adherence. [17]

The purpose of this study was to evaluate the effectiveness of an innovative patient educational program focused on risk counseling, designed to facilitate patient adherence to statin prescriptions. A secondary purpose was to determine whether the program would be more effective with patients who take action proactively to preserve their health.

## Methods

### Study design

A controlled trial was designed to test the effectiveness of the *Heart Health Counts (HHC) *program. This patient education program was designed to increase clarity in the cardiovascular risk dialogue, in order to facilitate statin adherence. The primary end point was adherence to filling statin prescriptions during a 120-day period by patients new to statin therapy.

To enroll, patients were asked to complete a consent form, and to complete a survey. Patients were enrolled at the time of a visit to their physician and were eligible to participate if they were at least 40-years of age and receiving a new prescription for statin therapy. Survey items of attitudes and beliefs related to cardiovascular disease and cholesterol management were included.

Identifying patients according to their attitudes concerning health and lifestyle change has been shown to be important in developing strategies to facilitate behavioral change [16].

Based on their survey responses, patients were categorized into three segments according to their health and medication attitudes: patients who regularly take action to preserve their health, patients committed to their health but not consistently taking action, and patients who do less to preserve health.

Data on statin prescription use from a national pharmacy claims database were used to determine whether or not patients achieved 120 day adherence. Based on region, prescription, age and gender, a control group of similar patients not exposed to the patient education intervention were identified from the national pharmacy claims database. The Western Institutional Review Board approved the study in October of 2006. Patients and physicians completed a consent form agreeing to participate. Physicians also completed a brief survey on their perceptions of the program following use of the brief counseling materials with their patients.

### Eligibility criteria

Physicians were eligible to participate if they provided adult primary care or were cardiologists. Physicians were required to read the protocol, to complete a brief online module concerning counseling patients with high cholesterol, and to complete a consent form and letter of agreement. Patients were eligible to participate if they are 40 years of age or older, had received a prescription for a lipid-lowering agent within the past 30 days, had not previously been on a cholesterol-lowering medicine within the past 12 months, were literate in English, and able to give written consent to receive patient educational mailings.

Power calculations indicated, within 95% confidence intervals, a sample including at least 200 patients (including 50 in each of two specific segments, those characterized as Health Preservers and those characterized as Health Commiteds), a total of 100 patients was needed to detect a 5% difference in adherence to statins at 120 days comparing segments to a control group. In order to determine the number of patients required for enrollment, it was estimated that each of the two segments of patients who were ready to act to preserve their health were representative of less than 10% of the total population, and a smaller percentage of these would actually fill their prescriptions. It was further estimated that 200 physicians were needed to each recruit approximately 10 patients with hypercholesterolemia new to statin therapy, a total of 2000 patients. Physicians were each allowed to recruit no more than 15 patients in order to ensure balanced representation among the physicians recruited. Reflecting the 95% confidence intervals, the significance level was set at .05.

### Segmenting patients

Five questions from the 20-question survey were used to segment patients into three groups. These questions were used to create segments, but were not validated for this purpose. Patients were asked to respond to the following statements using a 7-point agreement scale: 1) I feel tired most of the time 2) I feel older than I should 3) I do not care what is in the medication, just that it works 4) I am worried about the long-term side effects of medications and 5) Taking medication makes me feel like I'm doing everything I can to make myself better. Health Preservers were more likely to agree with question #5 and less likely to agree with questions 1,2,3, and 4. Health Committeds were more likely to agree with question 3 but less likely to agree with questions 1, 2 and 5. Others, those patients who were less likely to focus on health preservation, were more likely to agree with questions 1, 2, and 4, and less likely to agree with questions 3 or 5.

### Adherence measures

Data from a national pharmacy claims database were used to determine adherence to filling statin prescriptions. The average number of days for which statin prescriptions were written was 30 days. However, the number of days for which statin prescriptions were filled varied between sites and depended on the site's current supplies. Therefore, days of filled statin prescriptions were used in this study, rather than the number of prescriptions filled. Two adherence measures were calculated: 1) the average number of days of prescriptions filled for statins per patient during a 120-day time period, and 2) the percentage of patients who filled prescriptions for 120 days of statin therapy.

### Intervention

The *HHC *program provided tools to physicians to increase clarity in the cardiovascular risk dialogue, in order to facilitate statin adherence. The program was designed to facilitate an enhanced patient-physician dialogue without being labor-intensive for physicians and other healthcare providers. Physicians participating in this study received a counseling kit including 1) a set of 1-minute health manager patient education tools used to describe cholesterol risks, 2) patient contracts or pledges designed to confirm a patient's commitment to the prescribed medical regimen, 3) a copy of the National Cholesterol Education Program pocket guidelines and 4) a set of chart stickers. The materials were designed to address cardiovascular risk and to provide a context for patients who might be presented with a series of lab values but might not have a context for the severity of the risk. The 1-minute health manager and other *HHC *materials used color coding with green, yellow, and red to represent cardiovascular risk stratification, and to serve as a call to action for patients in the yellow and red zones. By fostering a more constructive patient-provider dialogue, a context could be created for a patient-physician partnership in developing achievable patient goals to reduce the risk of cardiovascular disease.

Following the office visit, patients new to statin therapy received 5 *HHC *mailings over a 4-month period. The four-color print mailings focused on various aspects of cardiovascular health and cardiovascular risk.

### Analysis

Descriptive statistics for categorical data were expressed as percentages; for the continuous data, the descriptive statistics were expressed as means (standard deviations). T-tests were used to compare the average numbers of days statin prescriptions were filled, and the percentage of patients who completed 120 days of statin therapy. On questions 1 and 4, a reflect and square root transformation was performed to normalize a distribution that differs only moderately from normal. The deviation is more substantial for questions 3 and 5, so we performed a reflect and inverse transformation. Comparisons of segmented groups were quantified by one-way multivariate analysis of variance (MANOVA), followed by post-hoc pairwise comparisons using the Student-Newman-Keuls (SNK) test and the Dunnett T3 test for data sets with unequal variances. Statistical significance was established as *p *< 0.05. All analyses were conducted using SPSS 16.0 (SPSS, Inc. Chicago, IL).

## Results

**Figure 1:**
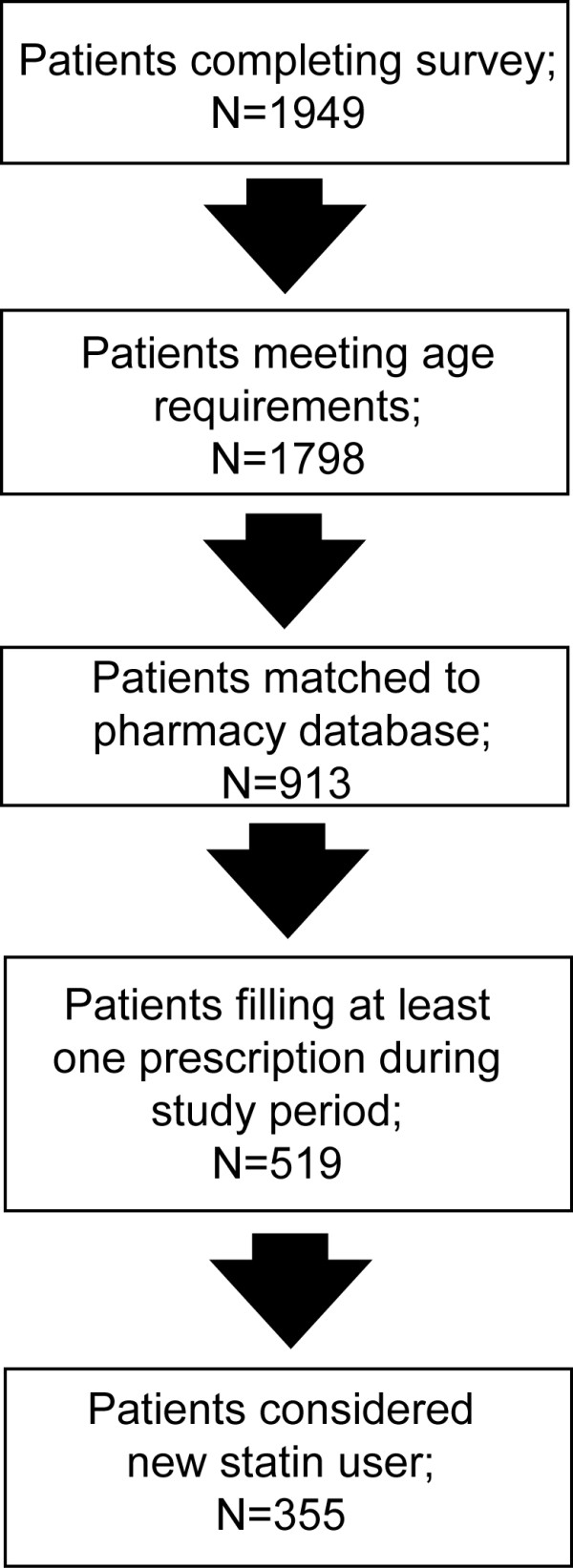
**Flow Chart of Patient Enrollment and Exclusion**. A total of 1949 patients were enrolled in this study and completed a survey. For overall analysis, only patients that met the age criteria (40 and above) were included. Further exclusions were made to analyze new statin patients that had filled at least prescription during the study period.

Patients were recruited for this study by 234 physicians from 39 states. The specialties of participating physicians were family medicine (59%), general internal medicine (37%), and cardiology (4%). A total of 1,949 patients consented, enrolled, and completed a survey. Of these, 1798 met the age requirement of 40 or more years of age (Figure 1). Patient data were de-identified for the purpose of obtaining pharmacy claims data. The average age of the patients was 58 years; 24.8% were between 40 and 50 years of age, 34.0% between 50 and 60 years, 23.9% between 60 and 70 years, and 17.3% over the age of 70. When asked about how they would rate their health overall on a scale of 1–7 with 1 representing "poor" and 7 representing "excellent," the average rating for the patient group was 4.92.

Nearly all of the patients surveyed felt their physicians listened carefully to them and explained things in a way they could understand. Most reported their physicians had talked to them about their current cholesterol levels, the health risks of high cholesterol, and about drug treatments to lower cholesterol. However, 159 (8.8%) reported "no" or "unsure" when asked whether their physician talked to them about setting goals for reducing cholesterol, and 246 (13.6%) would not usually raise concerns when their doctor does not ask about them. (Table 1)

**Table 1:** Physician patient communication by segment

**Questions**	**Health Preservers**			**Health Committeds**			**Others**			**Total**		
	**yes**	**no**	**unsure**	**yes**	**no**	**unsure**	**yes**	**no**	**unsure**	**yes**	**no**	**unsure**

1. Has your physician talked to you about drug treatments to lower your cholesterol?	204 (97.6)	4 (1.9)	1 (0.5)	318 (97.5)	5 (1.5)	3 (0.9)	1231 (97.5)	18 (1.4)	14 (1.1)	1753 (97.5)	27 (1.5)	18 (1.0)

2. Has your physician talked to you about your current cholesterol levels?	205 (98.1)	3 (1.4)	1 (0.5)	319 (97.9)	7 (2.1)	0 (0)	1240 (98.2)	18 (1.4)	5 (0.4)	1764 (98.1)	28 (1.6)	6 (0.3)

3. Did your physician talk to you about health risks of high cholesterol?	203 (97.1)	4 (1.9)	2 (1.0)	318 (97.5)	5 (1.5)	3 (0.9)	1210 (95.8)	30 (2.4)	23 (1.8)	1731 (96.2)	39 (2.1)	28 (1.6)

4. Has your physician talked to you about your cholesterol goals?	195 (93.3)	6 (2.9)	8 (3.8)	301 (92.3)	13 (4.0)	12 (3.7)	1143 (90.5)	64 (5.1)	56 (4.4)	1639 (91.2)	83 (4.6)	76 (4.2)

5. Do you feel your doctor has listened carefully to you?	202 (96.7)	1 (0.5)	6 (2.9)	323 (99.1)	2 (0.6)	1 (0.3)	1223 (96.8)	10 (0.8)	30 (2.4)	1639 (97.2)	13 (0.7)	37 (2.1)

6. Do you feel your doctor has explained things in a way you could understand?	201 (96.2)	2 (1.0)	6 (2.9)	320 (98.2)	3 (0.9)	3 (0.9)	1226 (97.1)	11 (0.9)	26 (2.1)	1747 (97.2)	16 (0.8)	35 (1.9)

7. Do you usually raise your concerns even when your doctor does not ask?	186 (89.0)	13 (6.2)	10 (4.8)	288 (88.3)	24 (7.4)	14 (4.3)	1078 (85.4)	124 (9.8)	61 (4.8)	1552 (86.3)	161 (8.9)	85 (4.7)

* Percentages of responses are in parentheses.

Of these enrolled patients, 913 were matched to the claims database of pharmacy claims (Figure 1); 519 filled at least one prescription during the treatment period of the study (Table 2). However, of the 519 patients who matched the claims database and filled at least one prescription, 164 patients had filled a statin prescription during the 12-month period before the initiation of the study and were considered ineligible as a new statin user. Around half of segment 1, Health Preservers, filled a new statin prescription (46.9%). Table 3 represents the average number of statin prescription days filled by participants according to segments and the percentage of patients who completed 120 days of new statin therapy. *HHC *participants achieved 12.4 more days on average of new statin prescriptions filled compared to the control group (*p *= .01). *HHC *participants were more likely to complete 120 days of statin therapy than similar patients who did not participate (*p *= .01). Patients in the Health Preservers segment were more likely (although not statistically significantly) to fill prescriptions for 120 days of statin therapy than other patients participating in the program and significantly more likely to fill prescriptions for the 120 days of therapy compared with similar patients who did not participate in the program (*p *= .01).

**Table 2:** Patients matched to pharmacy claims database by segment

**Patients By Segment**	**Matched to Claims Data^1^** **(N)**	**New Statin Script Filled^2^** **(N)**	**Matched Patients Filling Statin Script** **(%)**
Health Preservers	98	46	46.9%

Health Committeds	165	66	40.0%

Others	650	243	37.4%

Total *Heart Health Counts *Patients	913	355	38.9%

^1 ^Patients who had previously filled prescriptions in pharmacies associated with the national pharmacy claims database were matched to the claims database; they did not, however, fill a statin prescription during the study period at a pharmacy covered by the claims database.

^2 ^Patients who filled a new statin prescription in pharmacies associated with the national pharmacy claims database

**Table 3:** Days of new statin prescriptions filled

**Enrolled Patient Segments**	**N**	Mean # Days*	**p value**	% Completing 120 days*	**Segments Compared to Control** **p value**
Health Preservers	46	87.2	0.01	72.8	0.01

Health Committeds	66	77.5	0.05	65.3	0.05

Others	243	80.9	<0.01	67.5	0.01

All *Heart Health Counts *Patients	355	81.1	<0.01	67.8	<0.01

All Control Patients	196	68.7	-	57.8	-

Table 4 represents patient attitudes concerning cardiovascular risk related to elevated cholesterol. Utilizing one-way MANOVA, we determined that the Health Preserver, Health Committed, and other combined groups responded significantly differently to questions concerning cholesterol risk. Post hoc analysis using the SNK test revealed that Health Committed patients were significantly less likely to be concerned about their cholesterol level than the Health Preservers or other combined groups. However, both Health Committeds and Health Preservers groups were less confident about their ability to manage cholesterol levels than the other population segments. Based on the Dunnett T3 analysis, health committed patients differ from the other groups on whether high cholesterol is linked to heart attacks. All three groups significantly differed in their responses to whether taking medication every day to lower cholesterol is worth it to lower chances of a future heart attack. The Health Committeds group was most likely to be 1) concerned about cholesterol levels 2) to believe that cholesterol makes a difference in their overall health and 3) believe that people with high cholesterol are more likely to have a heart attack than people with low cholesterol. Both the Health Committeds and Health Preservers groups were more confident they could do what was needed to manage their cholesterol and to believe that taking medicine very day to lower cholesterol is worth it to lower risks of a future heart attack.

**Table 4:** Patient attitudes concerning cardiovascular risk related to elevated cholesterol

**Question**		**N**	**%**	**Mean**	**SD**	**F**	**P**	***η***_***p***_^**2**^	**SNK**	**Dunnett T3**
How concerned are you about your cholesterol level?	**P** **C** **O** **Total**	209 326 1263 1798	11.6 18.1 70.3 100.0	5.83 6.19 5.75 5.84	0.089 0.065 0.036 0.030	18.766	<0.001	0.004	C<P C<R	P≠C C≠R

My cholesterol level does not make a big difference to my total overall health.	**P** **C** **O** **Total**	209 326 1263 1798	11.6 18.1 70.3 100.0	2.84 3.30 2.98 3.02	0.149 0.138 0.059 0.051	3.662	0.026	0.004		

People with high cholesterol are more likely to have a heart attack than people with low cholesterol.	**P** **C** **O** **Total**	209 326 1263 1798	11.6 18.1 70.3 100.0	6.23 6.44 6.14 6.20	0.090 0.061 0.035 0.029	15.048	<0.001	0.016		C≠other

I am confident I can do what is needed to manage my cholesterol.	**P** **C** **Other** **Total**	209 326 1263 1798	11.6 18.1 70.3 100.0	6.03 6.03 5.71 5.81	0.087 0.073 0.036 0.031	14.907	<0.001	0.016	P < R C < R	P ≠ other C ≠ other

Taking medicine every day to lower cholesterol is worth it to me to lower the chances of having a heart attack in the future.	**P** **H** **O** **Total**	209 326 1263 1798	11.6 18.1 70.3 100.0	6.40 6.62 6.26 6.34	0.079 0.047 0.032 0.026	19.812	<0.001	0.022		P≠C C≠R

P = Health Preservers

C = Health Committeds

O = Others

One hundred and sixty-four physicians who recruited patients returned surveys after they used the *HHC *counseling kits in their office. Of these 93.3% felt the kits were easy to use; 87.8% felt the kit allowed them to clearly explain cardiovascular risk factors to their patients and 62.2% felt the kit helped their patients make positive lifestyle changes. Participating physicians identified the 1-minute cardiovascular risk manager for brief counseling as the most useful tool provided in the counseling kit (Table 5), followed by the *NCEP ATP III Pocket Guidelines*.

**Table 5:** Ratings of usefulness of *Heart Health Counts *kit components N = 164

**Components**	**Least 1**	**2**	**3**	**4**	**Most 5**
One-Minute Health Manager Profile	0.6%	3.7%	21.5%	39.9%	34.4%

*Hearth Health Counts *Pledge	5.0%	20.5%	31.1%	26.1%	17.4%

Chart Stickers	18.6%	19.9%	28.0%	21.7%	11.8%

*NCEP ATP III Pocket Guidelines*	3.7%	2.5%	19.6%	39.9%	34.4%

## Discussion

While the use of statin medication has clinically proven benefits in the treatment of heart disease, one of the well-documented barriers to its use is the large number of patients who do not fill the prescription, or stop use within the first year. Improving adherence to a treatment plan may result from a better understanding by the individual of personal risk as well as of the disease process and the role of statin medication in reducing cardiovascular risk. The *HHC *program has focused on the communication of cardiovascular risk in the dialogue between patient and physician in order to motivate the patient to adhere to the prescribed medical regimen.

Patients participating in the *HHC *study filled more prescriptions than control patients who were not exposed to the program. Based on the research highlighting the difficulties associated with labor-intensive interventions [2,5], the program was designed to intensify the physician patient dialogue without becoming labor intensive. The *Heart Health Counts *program aimed at maximizing the limited amount of time a physician might spend with a patient during the patient encounter by providing a simple, well-designed 1-minute risk counseling tool. Physicians stressed the benefits of the 1-minute cardiovascular risk manage tool and a pocket cholesterol management guideline, reporting that these tools facilitated patient management.

Given the scarce resources available for increasing adherence, the concept of segmentation for health education based on health beliefs has the potential to identify those patients who will benefit most from additional counseling and reinforcement of the benefits from a given treatment plan. The *Heart Health Counts *program was most effective with those individuals classified as Health Preservers, a group whose beliefs included a commitment to actively preserving their health. Physician-patient communication may be enhanced when a physician better understands a patient's approach to his/her own care [18-20] More research is needed, however, to better understand how to best design tailored interventions.

This study was limited in several ways. The use of prescribing data from retail pharmacies as a data source presents a series of limitations. The claims database captured commercial pharmacy data, but did not capture all events related to patient pharmacy claims Patients may have filled prescriptions in some other setting, by mail, or not at all. Using a national claims database does not allow a complete study of nonadherence since not all patients are represented in the database. Patient prescribing data is deidentified and aggregated by segment; thus, prescription data could not be connected to survey data. Filling a prescription did not actually guarantee taking the medicine. In future studies, more robust measures of adherence could track actual medication use by recording the dates and times of medicine bottle opening with electronic caps.

The study was also limited by the study period, where adherence was measured at 120 days. A longer period of follow-up would provide a more in-depth look at adherence patterns. The current study was not designed to track actual exposure to the mailed materials, leaving open the research question concerning the effectiveness of whether tailored mailed patient education materials increase statin adherence. Future research to attempt to resolve this question could include the use of emerging technologies, such as Internet sites that would allow tracking exposure to educational messages.

## Conclusion

In conclusion, providing tools to physicians to improve the quality of the physician-patient dialogue around cardiovascular risk shows promise in addressing the important issue of facilitating long-term adherence to statin therapy. Tools that increase the clarity of risk communication but do not add significantly to the labor of healthcare professionals are those that are most valuable in daily clinical practice.

Little attention has been paid in medical school and residency training programs in teaching physicians the best ways to communicate cardiovascular risk and to motivate patients to adhere to the prescribed medical regimens that prevent cardiovascular disease. Providing physicians and other healthcare professionals with tools to make the dialogue during the patient encounter more effective and efficient may significantly enhance patient adherence. Strategies that provide follow up for patients through reminders may reinforce the messages from this dialogue.

Patients vary in their attitudes towards their health and in taking on a new medical regimen. One size does not fit all in patient education and counseling. Simple strategies that support the physician-patient dialogue and those that provide reminders to patients about the need to reduce cardiovascular risk may have a significant impact on improving patient adherence and preventing cardiovascular disease.

## Competing interests

The authors declare that they have no competing interests.

## Authors' contributions

LC, CH, NB, RS, and MA contributed to the design and implementation of the study. GS and SZ contributed to the statistical analysis of the data. All authors contributed to the overall data analysis, discussion and conclusions. All authors contributed to the preparation of the manuscript.

## Pre-publication history

The pre-publication history for this paper can be accessed here:


